# Supplementary material for: Emergence of distinct and heterogeneous strains of amyloid beta with advanced Alzheimer’s disease pathology in Down syndrome
Source: Acta Neuropathol Commun. 2021 Dec 27;9:201. doi: 10.1186/s40478-021-01298-0 (PMC8711167; doi:10.1186/s40478-021-01298-0)
Supplement: Supplementary file 1 — Additional file 1. Supplementary Table 1. Biological data and experimental inclusion of each case. [file 40478_2021_1298_MOESM1_ESM.docx]

**Emergence of distinct and heterogeneous strains of amyloid beta with advanced Alzheimer’s disease pathology in Down syndrome**

Alison M. Maxwell^1^, Peng Yuan^2^, Brianna M. Rivera^2^, Wilder Schaaf^3^, Mihovil Mladinov^4^, Vee P. Prasher^5,6^, Andrew C. Robinson^7^, William F. DeGrado^1^, Carlo Condello^2,8^*

^1^Department of Pharmaceutical Chemistry, Cardiovascular Research Institute, University of California, San Francisco, CA 94158; ^2^Institute for Neurodegenerative Diseases, Weill Institute for Neurosciences, University of California, San Francisco, CA 94158; ^3^Department of Physics & Astronomy, San Francisco State University, San Francisco, CA 94132; ^4^Memory & Aging Center, Weill Institute for Neurosciences, University of California, San Francisco, CA 94158; ^5^South Birmingham Community NHS Trust, Birmingham, UK; ^6^Liverpool John Moores University, Liverpool, UK; ^7^Division of Neuroscience & Experimental Psychology, Faculty of Biology, Medicine and Health, School of Biological Sciences, The University of Manchester, Salford Royal Hospital, Salford, UK; ^8^Department of Neurology, Weill Institute for Neurosciences, University of California, San Francisco, CA 94158.

*Corresponding author. Email: carlo.condello@ucsf.edu

**Additional file 1:** Supplementary table 1.

**Supplementary table 1.** Biological data and experimental inclusion of each case.

| Bank^[[1]](#footnote-1)^ | ID | Cohort | Age | Sex | PMI (h) | *APOE* | X_Aβ_ | X_tau_ | IHC^[[2]](#footnote-2)^ | PCA^[[3]](#footnote-3)^ | ELISA^[[4]](#footnote-4)^ | HTRF^**^ |
| --- | --- | --- | --- | --- | --- | --- | --- | --- | --- | --- | --- | --- |
| UW | 12664 | DS | 48 | F | 3 | 3/3 |  |  |  |  | x |  |
| UW | 11-0033 | DS | 59 | F | 8 |  | 4 | 3 | x |  |  |  |
| UW | 11-1450 | DS | 49 | M | 14 |  | 4 | 4 | x |  |  |  |
| UW | 09-0007 | DS | 57 | F | 4 | 3/4 | 4 | 4 | x |  | x |  |
| UW | 00-0584 | DS | 62 | F | 4 | 3/3 | 4 | 4 | x |  | x |  |
| UW | 09-1549 | DS | 27 | F |  |  | 0 | 1 | x | o |  |  |
| MIA | 18_01 | DS | 24 | M | 24 | 3/3 | 0 | 0 | x | o | x |  |
| MIA | 19_01 | DS | 51 | F | 8 | 3/3 | 4 | 4 | x |  | x |  |
| MIA | 19_03 | DS | 57 | M | 11 | 3/4 | 4 | 4 | x |  | x |  |
| UMD | 4273 | DS | 33 | M | 36 |  | 1 | 1 | x | x |  |  |
| UMD | 4335 | DS * | 28 | M | 26 | 4/4 | 2 | 2 | x | x | x | x |
| UMD | 5005 | DS * | 39 | F | 12 |  | 1 | 0 | x | x |  |  |
| UMD | 5277 | DS * | 19 | M | 26 | 3/4 | 1 | 1 | x | x | x | x |
| UMD | 5341 | DS * | 25 | M | 24 | 3/3 | 1 | 1 | x | x | x | x |
| UMD | 5713 | DS | 25 | M | 22 | 2/3 | 0 | 1 | x | o | x | x |
| UMD | 4530 | DS * | 47 | F | 7 | 3/4 | 4 | 4 | x | x | x | x |
| UMD | 4659 | DS * | 46 | F | 7 | 3/4 | 4 | 4 | x | x | x | x |
| UMD | 4785 | DS * | 55 | F | 25 | 3/3 | 3 | 3 | x | x | x | x |
| UMD | 4870 | DS * | 51 | F | 4 | 2/3 | 4 | 4 | x | x | x | x |
| UMD | 4904 | DS * | 40 | M | 10 | 3/3 | 1 | 0 | x | o | x | x |
| UMD | 5386 | DS * | 64 | M | 20 | 3/4 | 3 | 2 | x | x | x | x |
| UMD | 5510 | DS * | 65 | M | 10 | 3/3 | 4 | 4 | x | x | x | x |
| UMD | 5600 | DS | 57 | F | 6 | 3/3 | 3 | 4 | x | x | x | x |
| UMD | 5783 | DS | 41 | M | 15 | 3/4 | 1 | 1 | x | x | x | x |
| UMD | 6151 | DS | 57 | M | 5 | 3/3 | 4 | 4 | x | x | x | x |
| UMD | 2854 | DS * | 15 | M | 14 |  | 0 | 0 | x |  | x |  |
| UMD | 2135 | DS * | 2 | M | 12 |  |  |  | x |  | x |  |
| UMD | 1947 | DS * | 0 | F | 24 |  |  |  | x |  |  |  |
| UMD | 267 | DS | 0 | M | 28 |  |  |  | x |  |  |  |
| UMD | 570 | DS | 2 | M | 24 |  |  |  | x |  |  |  |
| UMD | 714 | DS * | 2 | M | 17 |  |  |  |  |  |  |  |
| UMD | 718 | DS * | 1 | F | 28 |  |  |  | x |  |  |  |
| UMD | 832 | DS * | 1 | M | 23 |  |  |  | x |  |  |  |
| UMD | 1267 | DS | 10 | M | 17 |  |  |  |  |  |  |  |
| UMD | 1276 | DS * | 13 | M | 25 |  |  |  |  |  |  |  |
| UMD | 1282 | DS * | 0 | F | 28 |  |  |  | x |  |  |  |
| UMD | 4204 | DS | 9 | F | 30 |  | 1 | 0 | x | o |  |  |
| UMD | 5301 | DS * | 3 | F | 11 |  |  |  |  |  |  |  |
| UMD | 5874 | ADNC | 63 | F | 25 | 3/3 | 1 | 2 | x |  | x | x |
| UMD | 6054 | ADNC | 76 | M | 15 | 3/4 | 1 | 3 | x |  | x | x |
| BCN | 665 | DS | 59 | F | 8 | 3/4 | 3 | 4 | x | x | x | x |
| BCN | 714 | DS | 36 | F | 12 | 3/3 | 2 | 2 | x | x | x | x |
| BCN | 907 | DS | 63 | M | 6 | 3/3 | 3 | 4 | x | x | x | x |
| BCN | 1028 | DS | 67 | F | 11 | 3/3 | 3 | 3 | x | x | x | x |
| BCN | 1335 | DS | 62 | F | 9 | 3/4 | 4 | 4 | x | x | x | x |
| BCN | 1469 | DS | 62 | M | 6 | 3/4 | 4 | 3 | x | x | x | x |
| BCN | 1468 | ADNC | 64 | M | 10 | 3/3 | 1 | 2 | x | o | x | x |
| BCN | 1679 | ADNC | 90 | F | 12 | 3/3 | 1 | 3 | x | x | x | x |
| BCN | 1858 | ADNC | 83 | F | 8 | 3/3 | 0 | 1 | x |  | x | x |
| BCN | 1937 | ADNC | 83 | F | 8 | 3/3 | 1 | 2 | x | x | x | x |
| BCN | 1949 | ADNC | 86 | M | 8 | 3/3 | 1 | 1 | x |  | x | x |
| BCN | 1870 | ADNC | 97 | F | 7 | 3/3 | 2 | 2 | x | x | x | x |
| UCI | 29-93 | DS | 47 | F | 5 | 3/3 | 3 | 2 | x | o | x |  |
| UCI | 43-93 | DS | 48 | M | 3 | 3/3 | 4 | 4 | x | x | x | x |
| UCI | 46-94 | DS | 62 | F | 3 | 3/3 | 4 | 4 | x | x | x | x |
| UCI | 23-95 | DS | 48 | F | 2 | 2/2 | 2 | 1 | x | x | x | x |
| UCI | 42-98 | DS | 55 | F | 5 |  | 4 | 4 | x | x | x | x |
| UCI | 30-00 | DS | 61 | M | 11 | 3/3 | 4 | 4 | x | x | x | x |
| UCI | 13-02 | DS | 46 | M | 6 | 2/3 | 4 | 3 | x | x | x | x |
| UCI | 23-04 | DS | 58 | M | 3 | 3/4 | 3 | 4 | x |  | x | x |
| UCI | 33-04 | DS | 50 | F | 5 | 3/4 | 3 | 4 | x | x | x | x |
| UCI | 7-05 | DS | 54 | M | 5 | 3/3 | 4 | 4 | x | x | x | x |
| UCI | 22-05 | DS | 63 | F | 19 | 3/3 | 4 | 4 | x | x | x | x |
| UCI | 30-05 | DS | 57 | F | 3 | 3/3 | 4 | 4 | x | x | x | x |
| UCI | 29-06 | DS | 45 | F | 3 | 3/3 | 4 | 4 | x | x | x | x |
| UCI | 35-06 | DS | 48 | F | 18 | 3/3 | 1 | 1 | x | x | x | x |
| UCI | 31-07 | DS | 52 | F | 4 | 2/4 | 3 | 3 | x | x | x | x |
| UCI | 8-08 | DS | 57 | F | 5 | 3/3 | 3 | 3 | x | x | x | x |
| UCI | 31-08 | DS | 49 | M | 2 | 3/3 | 4 | 3 | x | x | x | x |
| UCI | 32-08 | DS | 42 | F | 5 | 3/4 | 3 | 2 | x | x | x | x |
| UCI | 38-08 | DS | 63 | F | 3 | 3/3 | 4 | 4 | x | x | x | x |
| UCI | 42-08 | DS | 55 | M | 5 | 3/3 | 3 | 4 | x | x | x | x |
| UCI | 8-09 | DS | 70 | M | 5 | 3/3 | 3 | 3 | x |  | x | x |
| UCI | 35-09 | DS | 66 | F | 3 | 3/3 | 3 | 3 | x | x | x | x |
| UCI | 31-10 | DS | 62 | F | 2 | 3/3 | 3 | 3 | x | x | x | x |
| UCI | 1-11 | DS | 45 | F | 9 | 3/3 | 3 | 3 | x |  | x | x |
| UCI | 30-11 | DS | 66 | M | 4 | 3/4 | 2 | 3 | x | x | x | x |
| UCI | 33-12 | DS | 56 | M | 5 | 3/3 | 3 | 4 | x | x | x | x |
| UCI | 36-12 | DS | 56 | F | 4 | 3/3 |  |  | x |  | x | x |
| UCI | 10-13 | DS | 56 | M | 4 | 4/4 | 4 | 4 | x | x | x | x |
| UCI | 21-14 | DS | 50 | M | 4 | 2/3 | 4 | 4 | x | x | x | x |
| UCI | 2-15 | DS | 43 | M | 4 | 3/3 | 4 | 3 | x | x | x | x |
| UCI | 5-15 | DS | 51 | F | 3 | 3/3 | 2 | 0 | x |  | x | x |
| UCI | 27-15 | DS PT21 | 72 | M | 5 | 3/3 | 0 | 1 | x |  | x | x |
| UCI | 32-15 | DS | 49 | M | 6 | 3/3 | 4 | 4 | x | x | x | x |
| UCI | 48-15 | DS | 55 | F | 5 | 3/3 |  |  | x |  | x | x |
| UCI | 4-16 | DS | 70 | M | 4 | 3/3 | 3 | 3 | x | x | x | x |
| UCI | 3-17 | DS | 57 | M | 4 | 3/3 | 3 | 4 | x | x | x | x |
| UCI | 7-17 | DS | 47 | F | 7 | 3/3 | 4 | 4 | x | x | x | x |
| UCI | 31-17 | DS | 56 | F | 5 |  | 3 | 4 | x |  | x | x |
| UCI | 33-17 | DS | 62 | F | 7 | 3/3 | 2 | 2 | x | x | x | x |
| UCI | 39-17 | DS | 58 | M | 6 | 3/4 | 3 | 4 | x | x | x | x |
| UCI | 4-02 | AD | 83 | M | 3 | 3/4 | 3 | 4 | x | x | x | x |
| UCI | 12-12 | AD | 82 | F | 6 | 3/4 | 3 | 2 | x | x | x | x |
| UCI | 14-08 | AD | 86 | M | 4 | 3/3 | 2 | 2 | x | x | x | x |
| UCI | 21-06 | AD | 82 | M | 5 | 3/4 | 1 | 1 | x | x | x | x |
| UCI | 37-15 | AD | 87 | F | 4 | 3/4 | 3 | 2 | x | x | x | x |
| UCI | 46-16 | ADNC | 78 | M | 3 | 3/4 | 2 | 0 | x | x | x | x |
| UCI | 10-17 | ADNC | 66 | F | 4 | 3/4 | 0 | 2 | x | o | x | x |
| UCI | 7-03 | ADNC | 90+^[[5]](#footnote-5)^ | M | 4 | 3/3 | 2 | 1 | x | x | x | x |
| UCI | 18-08 | ADNC | 84 | F | 4 | 3/3 | 2 | 1 | x | x | x | x |
| UCI | 14-17 | ADNC | 89 | F | 6 | 2/3 | 0 | 0 | x | o | x | x |
| MTS | 163827 | DS | 59 | F |  |  |  |  | x | o |  |  |
| MTS | 927140 | ADNC | 94 | F | 7 | 3/3 | 0 | 0 | x | o | x |  |
| MTS | 503571 | ADNC | 81 | F | 23 | 2/3 | 0 | 0 | x | o | x | x |
| KCL | A184/89 | DS | 47 | F | 24 |  |  |  |  |  | x |  |
| KCL | A185/89 | DS | 46 | M | 72 | 3/3 | 4 | 4 | x | x | x |  |
| KCL | A048/78 | DS | 42 | F |  |  | 1 | 1 | x | x |  |  |
| KCL | A046/88 | DS | 19 | F |  |  | 0 | 0 | x |  |  |  |
| KCL | A300/72 | DS | 21 | M | 21 |  | 0 | 0 | x | o |  |  |
| KCL | A585/80 | DS | 41 | M | 48 |  | 2 | 1 | x | x |  |  |
| KCL | A025/83 | DS | 38 | M | 48 |  | 1 | 0 | x | o |  |  |
| KCL | A027/83 | DS | 41 | M | 24 |  | 1 | 0 | x | x |  |  |
| KCL | A182/90 | DS | 65 | M | 9 | 3/3 | 4 | 4 | x | x | x |  |
| KCL | A246/91 | DS | 54 | M | 9 | 2/4 | 4 | 4 | x | x | x |  |
| KCL | A300/95 | DS | 63 | M | 120 | 3/4 | 3 | 3 | x | x | x |  |
| KCL | A161/09 | DS | 56 | F | 29 | 3/4 | 4 | 4 | x | o | x |  |
| KCL | A077/16 | DS | 37 | M | 48 | 3/3 | 1 | 0 | x | x | x |  |
| KCL | A372/18 | DS | 54 | F | 20 |  | 4 | 4 | x | o | x |  |
| KCL | A283/98 | AD | 40 | M | 97 | 3/3 | 0 | 0 | x | o | x |  |
| KCL | A166/04 | AD | 43 | M | 28 | 3/4 | 4 | 4 | x | x | x |  |
| KCL | A061/03 | AD | 55 | M | 18 |  | 4 | 4 | x | x | x |  |
| KCL | A085/98 | AD | 55 | M | 27 |  | 4 | 4 |  | x | x |  |
| KCL | A067/02 | AD | 57 | M | 43 |  | 4 | 4 | x | x | x |  |
| KCL | A035/03 | AD | 59 | F | 36 |  | 4 | 4 | x | x | x |  |
| KCL | A348/94 | AD | 59 | M | 5 | 3/3 | 1 | 0 | x | o | x |  |
| KCL | A228/99 | AD | 59 | F | 58 |  | 4 | 4 | x | x | x |  |
| MCR | BBN_2964 | DS | 62 | F |  |  |  |  | x |  |  |  |
| MCR | BBN_2965 | DS | 60 | M |  |  |  |  | x |  |  |  |
| MCR | BBN_2966 | DS | 13 | M |  |  |  |  | x | o |  |  |
| MCR | BBN_2967 | DS | 62 | F |  |  |  |  | x |  |  |  |
| MCR | BBN_2968 | DS | 53 | M |  |  |  |  | x |  |  |  |
| MCR | BBN_2969 | DS | 64 | M |  |  |  |  | x |  |  |  |
| MCR | BBN_2973 | DS | 37 | F |  |  | 1 | 0 | x | x |  |  |
| MCR | BBN_2974 | DS | 58 | M |  |  |  |  | x |  |  |  |
| MCR | BBN_2975 | DS | 65 | M |  |  |  |  | x |  |  |  |
| MCR | BBN_2978 | DS | 57 | M |  |  |  |  | x |  |  |  |
| MCR | BBN_2981 | DS | 50 | M |  |  |  |  | x |  |  |  |
| MCR | BBN_2984 | DS | 64 | M |  |  |  |  | x |  |  |  |
| MCR | BBN_2985 | DS | 71 | M |  |  |  |  | x |  |  |  |
| MCR | BBN_2987 | DS | 58 | F |  |  |  |  | x |  |  |  |
| MCR | BBN_2990 | DS | 60 | M |  |  |  |  | x |  |  |  |
| MCR | BBN_2996 | AD | 60 | M |  | 3/4 |  |  | x |  |  |  |
| MCR | BBN_3011 | DS | 38 | M |  |  | 3 | 4 | x | x |  |  |
| MCR | BBN_3012 | DS | 58 | F |  |  |  |  | x |  |  |  |
| MCR | BBN_3020 | DS | 58 | F |  |  | 4 | 4 | x | x |  |  |
| MCR | BBN_3021 | DS | 56 | M |  |  | 4 | 4 | x | x |  |  |
| MCR | BBN_3022 | AD | 71 | F |  |  | 4 | 3 | x | x |  |  |
| MCR | BBN_3057 | AD | 53 | F |  | 3/3 | 4 | 4 | x | x |  |  |
| MCR | BBN_3072 | AD | 44 | M |  |  | 4 | 4 | x | x |  |  |
| MCR | BBN_3112 | DS | 9 | M |  |  |  |  | x | o |  |  |
| MCR | BBN_3118 | DS | 61 | F |  |  |  |  | x |  |  |  |
| MCR | BBN_3202 | AD | 50 | M |  | 2/3 |  |  | x |  |  |  |
| MCR | BBN_3223 | AD | 65 | F |  | 3/4 |  |  | x |  |  |  |
| MCR | BBN_3224 | DS | 9 | F |  |  |  |  | x |  |  |  |
| MCR | BBN_3252 | AD | 79 | F |  |  |  |  | x | o |  |  |
| MCR | BBN_3253 | AD | 64 | M |  | 2/3 | 4 | 4 | x |  |  |  |
| MCR | BBN_3263 | AD | 56 | M |  |  | 3 | 4 | x | x |  |  |
| MCR | BBN_3269 | AD | 66 | M |  |  | 3 | 3 | x | x |  |  |
| MCR | BBN_3352 | DS | 61 | F |  |  |  |  | x | o |  |  |
| MCR | BBN_3353 | DS | 58 | F |  | 2/3 |  |  | x |  |  |  |
| MCR | BBN_3354 | DS | 62 | M |  | 3/3 |  |  | x |  |  |  |
| MCR | BBN_3355 | DS | 58 | M |  | 3/4 |  |  | x |  |  |  |
| MCR | BBN_3356 | DS | 53 | F |  | 3/4 |  |  | x |  |  |  |
| MCR | BBN_3358 | DS | 60 | F |  | 3/3 |  |  | x |  |  |  |
| MCR | BBN_3363 | DS | 59 | M |  | 3/3 |  |  | x |  |  |  |
| MCR | BBN_3364 | DS | 61 | M |  | 3/3 |  |  | x |  |  |  |
| MCR | BBN_3365 | DS | 55 | M |  |  |  |  | x |  |  |  |
| MCR | BBN_3380 | AD | 65 | M |  | 3/3 | 3 | 2 | x |  |  |  |
| MCR | BBN_3417 | AD | 45 | M | 79 | 3/3 | 3 | 3 | x |  |  |  |
| MCR | BBN_3437 | DS | 58 | M |  |  |  |  | x |  |  |  |
| MCR | BBN_3438 | DS | 56 | F |  | 3/3 |  |  | x |  |  |  |
| MCR | BBN_3439 | DS | 62 | M |  | 3/4 |  |  | x |  |  |  |
| MCR | BBN_3440 | DS | 65 | F |  | 3/3 |  |  | x |  |  |  |
| MCR | BBN_3441 | DS | 62 | F |  |  |  |  | x |  |  |  |
| MCR | BBN_24361 | AD | 63 | F | 54 |  | 4 | 4 | x |  |  |  |
| MCR | BBN_24555 | AD | 61 | F | 102 |  | 3 | 4 | x |  |  |  |
| MCR | BBN005.28400 | AD | 59 | F | 87 |  | 4 | 4 | x |  |  |  |
| MCR | BBN005.32913 | AD | 66 | F | 51 |  | 3 | 4 | x |  |  |  |
| MCR | BBN_14416 | ADNC | 89 | M | 48 | 2/3 | 1 | 2 | x | x |  |  |
| MCR | BBN_3446 | ADNC | 92 | F | 37 |  | 2 | 2 | x | o |  |  |
| MCR | BBN_14792 | ADNC | 90 | F | 78 | 3/3 | 1 | 0 | x | o |  |  |
| MCR | BBN_24212 | ADNC | 82 | F | 75 |  | 2 | 2 | x | x |  |  |
| MCR | BBN_24350 | ADNC | 76 | F | 98 |  | 1 | 1 | x | x |  |  |
| MCR | BBN_25922 | ADNC | 90 | F | 103 |  | 1 | 2 | x | x |  |  |
| MCR | BBN005.32526 | ADNC | 101 | F | 136 |  | 1 | 1 | x | x |  |  |
| HVD | AN02218 | DS | 51 | M | 16 | 3/3 |  |  |  |  | x |  |
| HVD | S19497 | ADNC | 93 | F | 18 | 3/3 | 0 |  | x | o | x |  |
| OXF | BBN004.34199 | AD | 103 | F | 35 | 2/3 | 3 | 4 | x |  | x | x |
| OXF | BBN004.26244 | AD | 75 | M | 46 | 3/4 | 3 | 4 | x | x | x | x |
| OXF | BBN004.26238 | AD | 87 | F | 68 | 3/4 | 3 | 4 | x | x | x | x |
| OXF | BBN_19683 | AD | 90 | F | 48 | 3/3 | 3 | 4 | x | x | x | x |
| OXF | BBN004.32861 | AD | 80 | F | 78 | 3/3 | 3 | 4 | x | x | x | x |
| OXF | BBN004.28928 | DS | 32 | M | 96 |  | 1 | 2 | x | x |  |  |
| OXF | BBN004.28911 | DS | 7 | F | 72 |  | 0 | 0 | x | o |  |  |
| OXF | BBN004.26750 | DS | 35 | M |  |  | 1 | 1 | x | o |  |  |
| OXF | BBN004.26748 | DS | 11 | M | 10 |  | 0 | 0 | x |  |  |  |
| OXF | BBN004.26647 | DS | 13 | F |  |  | 1 |  | x | o |  |  |
| OXF | BBN004.26645 | DS | 22 | M |  |  |  |  | x |  |  |  |
| OXF | BBN004.26644 | DS | 39 | F | 95 |  |  |  | x | o |  |  |
| OXF | BBN004.26635 | DS | 36 | M |  |  | 2 | 2 | x | x |  |  |
| OXF | BBN004.26632 | DS | 27 | F | 86 |  | 0 |  | x | o |  |  |
| OXF | BBN004.26630 | DS | 42 | F |  |  | 1 | 0 | x | x |  |  |
| OXF | BBN004.26628 | DS | 42 | M | 72 |  | 2 | 1 | x | x |  |  |
| NYU | 00-059 | DS | 33 | unk |  |  | 2 | 2 | x | o |  |  |
| NYU | 01-035 | DS | 23 | unk |  |  |  |  | x |  |  |  |
| NYU | 11-233 | DS | 47 | unk |  |  |  |  | x |  |  |  |
| NYU | 16-245 | DS | 48 | unk |  |  |  |  | x |  |  |  |
| NYU | 18-052 | DS | 58 | unk |  |  |  |  | x |  |  |  |
| UCSF | 2509 | ADNC | 103 | M |  | 3/3 | 2 | 2 | x | x |  |  |

1. Abbrevviations: UW = University of Washington; UMD = University of Maryland; MIA = University of Miami; BCN = IDIBAPS (Barcelona); MTS = Mt Sinai; HVD = Harvard University; MCR = Manchester; KCL = King’s College University; UCI = University of California, Irvine; NYU = New York University

   * Karyotype-confirmed DS [↑](#footnote-ref-1)
2. x = method performed on this sample and included in results [↑](#footnote-ref-2)
3. x = included in PCA; o = inclusion was attempted but spectra could not be detected or analyzed [↑](#footnote-ref-3)
4. ELISA was used to measure APP and soluble tau concentrations; HTRF was used to measure Aβ and insoluble tau species concentrations [↑](#footnote-ref-4)
5. This case is approximated as 90 years old in our analyses. [↑](#footnote-ref-5)
