# Supplementary material for: Emergence of distinct and heterogeneous strains of amyloid beta with advanced Alzheimer’s disease pathology in Down syndrome
Source: Acta Neuropathol Commun. 2021 Dec 27;9:201. doi: 10.1186/s40478-021-01298-0 (PMC8711167; doi:10.1186/s40478-021-01298-0)
Supplement: Supplementary file 2 — Additional file 2. Supplementary Figures 1 through 4. [file 40478_2021_1298_MOESM2_ESM.docx]

**Emergence of distinct and heterogeneous strains of amyloid beta with advanced Alzheimer’s disease pathology in Down syndrome**

Alison M. Maxwell^1^, Peng Yuan^2^, Brianna M. Rivera^2^, Wilder Schaaf^3^, Mihovil Mladinov^4^, Vee P. Prasher^5,6^, Andrew C. Robinson^7^, William F. DeGrado^1^, Carlo Condello^2,8^*

^1^Department of Pharmaceutical Chemistry, Cardiovascular Research Institute, University of California, San Francisco, CA 94158; ^2^Institute for Neurodegenerative Diseases, Weill Institute for Neurosciences, University of California, San Francisco, CA 94158; ^3^Department of Physics & Astronomy, San Francisco State University, San Francisco, CA 94132; ^4^Memory & Aging Center, Weill Institute for Neurosciences, University of California, San Francisco, CA 94158; ^5^South Birmingham Community NHS Trust, Birmingham, UK; ^6^Liverpool John Moores University, Liverpool, UK; ^7^Division of Neuroscience & Experimental Psychology, Faculty of Biology, Medicine and Health, School of Biological Sciences, The University of Manchester, Salford Royal Hospital, Salford, UK; ^8^Department of Neurology, Weill Institute for Neurosciences, University of California, San Francisco, CA 94158.

*Corresponding author. Email: carlo.condello@ucsf.edu

**Additional file 2:** Supplementary figures 1 through 4.

| **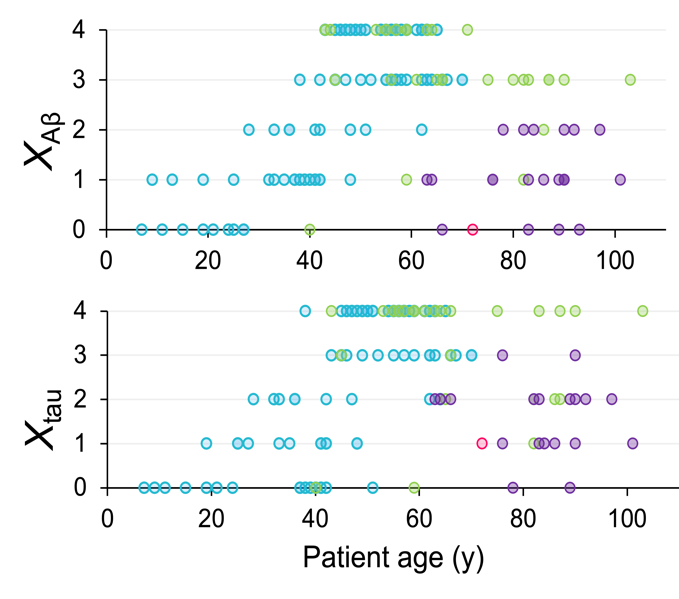** |
| --- |
| **Supplementary figure 1. Aβ and tau scores relative to patient age at death**. Scores were determined through manual evaluation of Aβ40, Aβ42, and pTau load in IHC-stained frontal cortical tissue sections. DS subjects are shown in blue, AD in green, ADNC in purple, and PT21 in pink. |

| **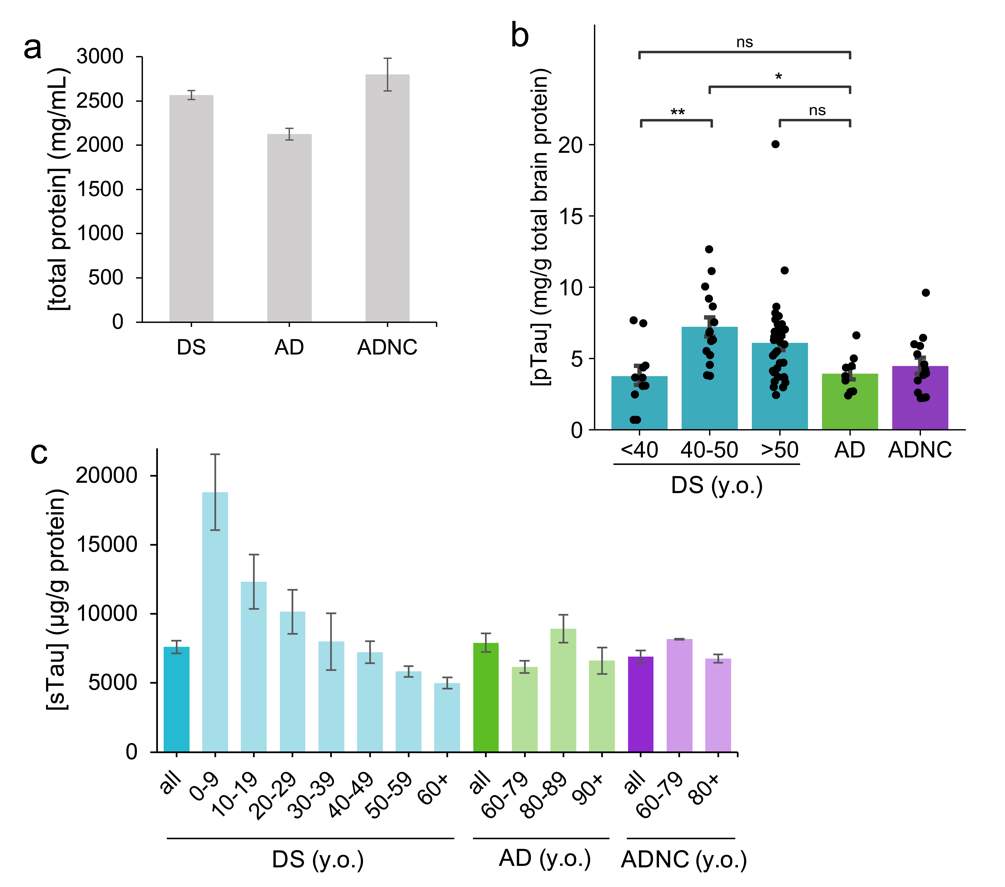** |
| --- |
| **Supplementary figure 2:** **Total protein concentrations per cohort and tau species concentrations in greater granularity.** (**A**) Total protein in 10% BH assayed by BCA (**B**) S203/T205 pTau concentrations as measured by HTRF delineated by age in DS, ± SEM. pTau is significantly higher in DS after 40 years of age compared to in younger DS individuals and compared to AD. Significance values were determined by ANOVA with Tukey's multiple comparison test. *p*-value: *: 0.01 < p ≤ 0.05; **: 0.001 < p ≤ 0.01, ***: 0.0001 < p ≤ 0.001. (**C**) Soluble tau concentrations as measured by ELISA decline with age in DS, ± SEM. |

| **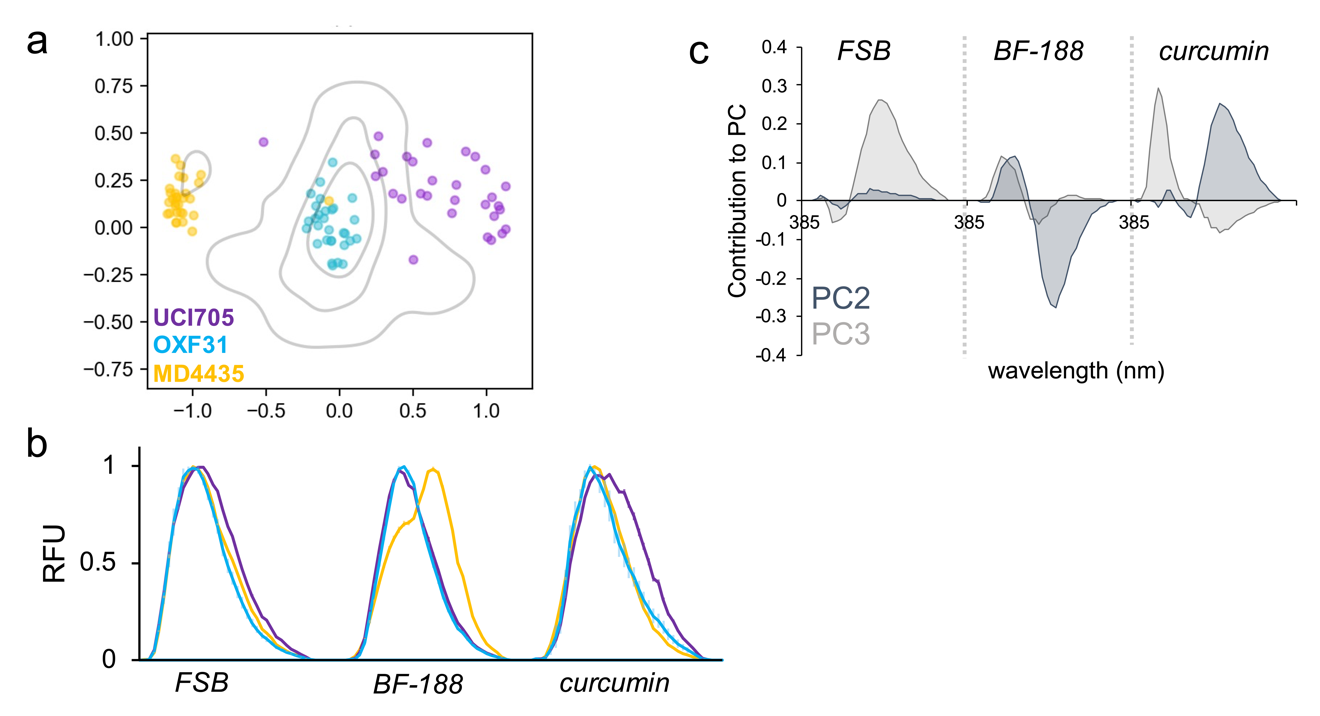** |
| --- |
| **Supplementary figure 3: Spectral contributions to the analysis.** (**A**) To provide examples of disparate patient spectra, plaques from three cases from different areas of the eigenspace are chosen. **(B)** The average spectral vector from each case in (A) is shown ± SEM (n=35-82 plaques). **(C)** The relative contribution of each wavelength of each dye to the principal component space. PC2 mostly reflects information from BF-188 and curcumin, whereas PC3 is predominately FSB and curcumin. |
|  |

| **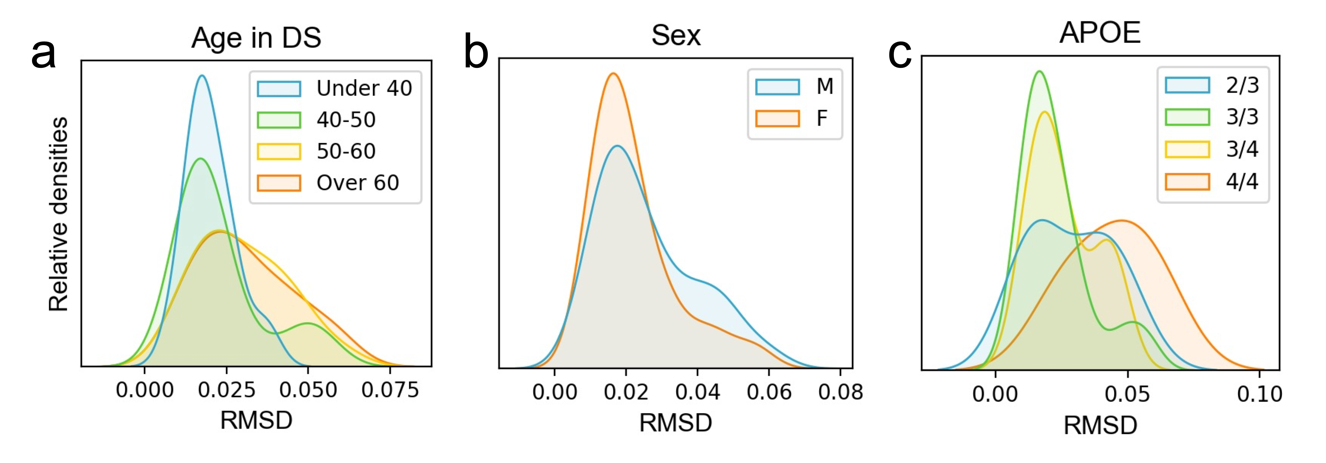** |
| --- |
| **Supplementary figure 4: Per-patient RMSD distributions by age, sex, and APOE.** (**A**) RMSD by DS patient age at death, (**B**) by sex, and (**C**) by APOE genotype, when known. RMSD values were calculated as the distances in PC2, PC3, and PC4 of each vector from the centroid of all the vectors for a given case. The relative densities are shown as a Gaussian KDE, normalized to the area under the curve, of the RMSDs calculated for all the cases in a given group. |
